# Application Value of Information-Based Health Education and Continuity of Care in Patients With Peptic Ulcer

**DOI:** 10.3389/fpubh.2021.694128

**Published:** 2021-09-01

**Authors:** Aihong Liu, Yuhua Kuang, Ruiping Huang, Qunying Ge

**Affiliations:** ^1^Department of Endoscopy, Jiaozhou Central Hospital of Qingdao, Qingdao, China; ^2^Department of General Medicine, Qingdao Jimo People's Hospital, Qingdao, China; ^3^Department of Infection Control, Qingdao Jimo People's Hospital, Qingdao, China; ^4^Hemodialysis Department, Jiaozhou People's Hospital, Qingdao, China

**Keywords:** information-based health education, continuity of care, peptic ulcer, SF-36, self-rating anxiety scale

## Abstract

**Objective:** This study is to assess the application value of information-based health education and continuity of care in patients with PU (peptic ulcer).

**Methods:** Patients (116) with PU who have been treated in the hospital between January 2019 and October 2020 were taken as research objects and equally assigned to a control group and an observation group in a random manner. In contrast to the routine care applied to the control group, the observation group received information-based health education and continuity of care intervention. The clinical efficacy, the mastery of health knowledge, self-care ability, medication compliance, quality of life, mental state, and nursing satisfaction of the two groups were compared.

**Results:** After the intervention, the total effective rate, health knowledge adequate rate, Exercise of Self-Care Agency (ESCA) scores of all dimensions, the MOS 36-item short-form health survey (SF-36) scores of all dimensions, medication compliance rate, and total nursing satisfaction of the observation group all notably exceeded those of the control group, with a *p* < 0.05. Patients of the group with continuity of care intervention showed lower Self-Rating Anxiety Scale (SAS) and Self-Rating Depression Scale (SDS) scores, as compared to the group with conventional care (*p* < 0.05).

**Conclusion:** Information-based health education and continuity of care elevates the medication adherence and nursing satisfaction of patients with PU, enhances disease-related knowledge of patients and their self-care ability, and eventually ameliorates the quality of life and psychological state. It is worthy of clinical application.

## Introduction

Peptic ulcer (PU), characterized by a long course of the disease and higher relapse, is a common disease in gastroenterology, among which gastric ulcer and duodenal ulcer are the most predominant ones (1). Insufficient disease-related knowledge and self-care ability, poor compliance after discharge, poor medication adherence, and the recurrence of the disease may hinder the quality of life of patients and give rise to negative mental states such as anxiety and depression, which takes a toll on clinical treatment (2–4). Therefore, it is essential to optimize the care schemes and ameliorate the quality of care for a better therapeutic effect of patients with PU. Continuity of care, usually referring to the continuation of care from hospital to home, is a new and humane nursing mode (5–8). Traditional health education, predominantly featured with oral education and paper publicity, is undermined by its humdrum education form, fragmented and insipid content, lack of innovation and flexibility, which leads to poor grasp of knowledge of the patients and an undesirable education result (9). With the rapid development of information technology, information-based health education, with flexible and diverse forms of education and comprehensive content, is increasingly and widely used in clinical practice. It has achieved promising results in the nursing process of various chronic diseases (10). At present, whether information-based health education combined with the continuity of care is applicable in PU or not is poorly understood. Therefore, this study aims to provide a new idea for optimizing the nursing plan of patients with PU by analyzing the application effect of this nursing method in patients with PU.

## Materials and Methods

### General Information

Patients (116) with PU admitted to the hospital from January 2019 to October 2020 were recruited.

Inclusion criteria were as follows: (1) the patient met with the diagnostic criteria of PU (11); (2) the diameter of the ulcer was >2 cm and the number of ulcers did not exceed 2; (3) the patients did not tend to hemorrhage throughout the body; (4) the patients had no mental illness, no cognitive or communication disorders; (5) the information of the patients was true and complete; and (6) patients with acute bleeding.

Exclusion criteria were as follows: (1) patients with cancerous ulcer; (2) patients with malignant gastrointestinal bleeding; (3) patients with a history of gastrointestinal surgery; (4) patients with severe organ disease; and (5) patients with gastroesophageal varices.

This study was approved by the ethics committee of the hospital, and all patients were informed and signed a consent form.

Patients were equally assigned to a control group (58 cases) and an observation group (58 cases). Of 58 cases in the control group, there were 32 men and 26 women; they were aged between 35 and 68, with a mean age of 49.28 ± 5.49; their course of the disease varied from 5 months to 8 years, with a mean course of 5.12 ± 1.07 years; as for the type of ulcer, there were 17 cases of the gastric ulcer; 29 cases of duodenal bulb ulcer, and 12 cases of compound ulcer. There were 30 men and 28 women in the observation group; they were aged between 37 and 65, with a mean age of 50.06 ± 5.31; their course of the disease varied from 6 months to 9 years, with a mean course of 5.61 ± 1.23 years; type of ulcers: 18 cases of the gastric ulcer, 27 cases of duodenal bulb ulcer, and 13 cases of compound ulcer. No significant difference was found between the general information of the two groups (*p* > 0.05).

### Research methods

The ulcer surface before and after treatment was observed with an electronic gastroscope. Gastroscopy is currently the most common, direct, and reliable method for diagnosing PU, which can directly observe the stomach, duodenal mucosa, and its lesions, and retain image data; The diseased tissue can be taken under direct vision of gastroscopy for histomorphological examination and *Helicobacter pylori* detection, which can be used to distinguish benign and malignant ulcers. It can detect small and shallow ulcers that are difficult to locate on X-ray barium meal examination, with higher sensitivity and specificity. Moreover, gastroscopy can treat ulcer-related complications while discovering ulcers, such as the treatment of ulcer bleeding under endoscopy.

#### Routine Care

The control group received routine care. With reference to the Nursing Regulations Manual and Nursing Principles Document, during the hospitalization, patients were provided with basic care such as medical condition monitoring, medication guidance, dietary care, psychological care, rehabilitation care, and oral health knowledge education. After the discharge, the patients were informed of the daily precautions and required to take the medicine on time and to review regularly. The patients should seek medical attention if they did not feel well.

#### Information-Based Health Education Combined With the Continuity of Care

The observation group adopted information-based health education combined with the continuity of care. Specific steps were as follows: (1) Setting up a team: The team consisted of one attending physician, one head nurse, and six nurses. Each member had taken relevant training courses and mastered nursing skills. (2) Creating a platform: a group of patients with PU (WeChat or QQ group) should be set up, and a WeChat official account platform for health promotion and education should be established. (3) Information-based health education: The public account content was regularly updated and shared with the patient group; disease knowledge lectures both online and offline were held regularly; PU health knowledge manuals were distributed to the patients, which explained in detail the causes of disease, preventive measures, and other related knowledge; short videos of disease knowledge were shared to the patient group to vividly explain the disease-related knowledge; patients were encouraged to communicate and encourage each other. (4) Medication care: for intravenous injection, it was suggested to choose a site that did not affect the daily life of the patients. An indwelling needle could be set if necessary to reduce the number of punctures; during oral administration, the patients were addressed with the name of the drug, its effect, time of administration, method of administration, dosage, and possible adverse reactions. The patients and family members were instructed that if the patients suffered serious discomfort after taking the medicine, they should immediately inform the medical staff for treatment. (5) Environmental care: the ward needed to be clean, sanitary, and ventilated; the decoration was mainly in soft colors to create a comfortable atmosphere. (6) Psychological nursing: the nursing staff should treat the patients gently and smilingly, and actively communicate with the patients to fully understand the psychological state of the patients; the staff needed to comfort the patients, tell the patients the recovery cases, and help the patients eliminate their fear of disease to build up their confidence in treatment; the staff should orally instruct the patients to be patient as PU has a long course, take drugs, stay optimistic, and actively cooperate with the treatment. (7) Discharge guidance: health guidance was provided to patients on the day of discharge. After the discharge, the patients were instructed to avoid irritating foods such as raw, cold, and spicy, have more meals a day but less food at each, and try to eat nutritious and easy-to-digest foods; patients should pay attention to keeping the living environment clean and ventilated; the patients should develop good behavior habits; the patients should take medicines on time and correctly, and follow-up regularly to understand the recovery status. (8) Continuity of care: a follow-up patient archive was established to record in detail the residential address of the patients, contact information, WeChat account, and family contact information. After the discharge, the patients were urged to take medication on time from WeChat every day; telephone follow-up once a week was needed, or video follow-up if necessary; family follow-up was conducted once a month to understand the medication and recovery status of the patients. The doubts of the patients were answered on the spot, and the daily self-care precautions were explained to them for correction. Communication with the patients was indispensable to understand the mental state of the patients, and psychological counseling was provided if necessary.

### Indicator Observation

#### Clinical Efficacy

After an intervention of 3 months, the clinical efficacy of the two groups of patients was compared. (1) Significantly effective: the symptoms disappeared completely and the ulcer was covered with a white scar; (2) effective: the symptoms disappeared, the ulcer surface was reduced, or it was in the healing period; and (3) ineffective: the symptoms have not improved, the ulcer surface has not been reduced, or it was in the active stage. Total effective rate = cases of (significantly effective + effective)/total number of cases × 100% (12).

#### Mastery of Health Knowledge

The self-made PU health knowledge test was employed to test the patients. The analysis was conducted based on the results of the test to obtain the health knowledge level of the patients. The test contained a total of 10 items, each with 10 points and a full score of 100 points. Adequate: ≥80 points; inadequate: <80 points. Health knowledge adequate rate = number of adequate cases/total number of cases × 100%.

#### Self-Care Ability

After the intervention, the ESCA scale was used to evaluate the self-care ability of the two groups of patients. The ESCA scale involved four dimensions, namely self-concept (9 items, a total of 36 points), self-responsibility (8 items, a total of 32 points), self-care skills (12 items, a total of 48 points), and health knowledge level (14 items, 56 points in total), a total of 43 items. According to the 3-level scoring method, the total score was 0–172 points. The higher the score, the stronger the self-care ability of the patient. Low: 0–57 points; medium: 58–115 points; high: 116–172 points (13).

#### Medication Compliance

Based on the results of the medication compliance questionnaire by Morishy, the medication compliance of the two groups was compared. The total score was 0–8. The higher the score, the better the medication compliance. Complete compliance: 8 points; partial compliance: 6–7 points; and non-compliance: <6 points (14). Total compliance rate = cases of (complete compliance + partial compliance)/total number of cases × 100%.

#### Quality of Life

The MOS 36-item short-form health survey (SF-36) scale was used to evaluate the quality of life of the two groups of patients. There were a total of eight evaluation dimensions, namely, physical functioning (PF), role physical function (RP), body pain (BP), general health (GH), vitality (VT), social functioning (SF), role emotional (RE), and mental health (MH). The total score for each dimension was 0–100 points. The higher the score, the higher the quality of life of the patient (15).

#### Mental State

The mental state of the two groups of patients was assessed by using the Self-Rating Anxiety Scale (SAS) and Self-Rating Depression Scale (SDS). Both scales contained 20 items. Each item was scored on a scale of 1–4, with a total score of 0–100 points. In the SAS scale, normal: <50 points; mild anxiety: 50–59 points; moderate anxiety: 60–69 points; and severe anxiety >69 points. The higher the score, the more severe the anxiety symptoms. In the SDS rating scale, normal: <53 points; mild depression: 53–62 points; moderate depression: 63–72 points; and severe depression: ≥73 points (16).

#### Nursing Satisfaction

A self-made questionnaire on nursing satisfaction of patients with PU in the hospital was distributed to the two groups of patients, and the nursing satisfaction of the two groups of patients was compared. The questionnaire contained a total of 20 items, each with a score of 5 points, and a total of 100 points. The ratings were very satisfied, satisfied, and dissatisfied. Very satisfied: 91–100 points; satisfied: 70–90 points; and dissatisfied: <70 points. Total satisfaction = cases of (very satisfied + satisfied)/total number of cases × 100%.

### Statistical Processing

Data management and analysis were performed by using SPSS24.0. The measurement data were analyzed by using a *t*-test, represented by (x̄±s); the χ^2^ test is performed for the analysis of the count data, represented by [*n*(%)]. A statistical difference was determined when a *p* < 0.05.

## Results

### Comparison of Clinical Efficacy Between the Two Groups of Patients

As can be seen from Table 1 below, a higher total effective rate of the observation group stood out by comparing with the control group (*p* < 0.05).

**Table 1:** Comparison of clinical efficacy between the two groups of patients [*n* (%)].

**Groups**	**Significantly effective**	**Effective**	**Ineffective**	**Total effective rate**
Control group (*n* = 58)	15 (25.86)	36 (62.07)	7 (12.07)	51 (87.93)
Observation group (*n* = 58)	26 (44.83)	31 (53.45)	1 (1.72)	57 (98.28)
χ^2^				4.833
*P*				0.028

### Comparison of the health knowledge mastery of the Two groups of patients

**Figure 1:**
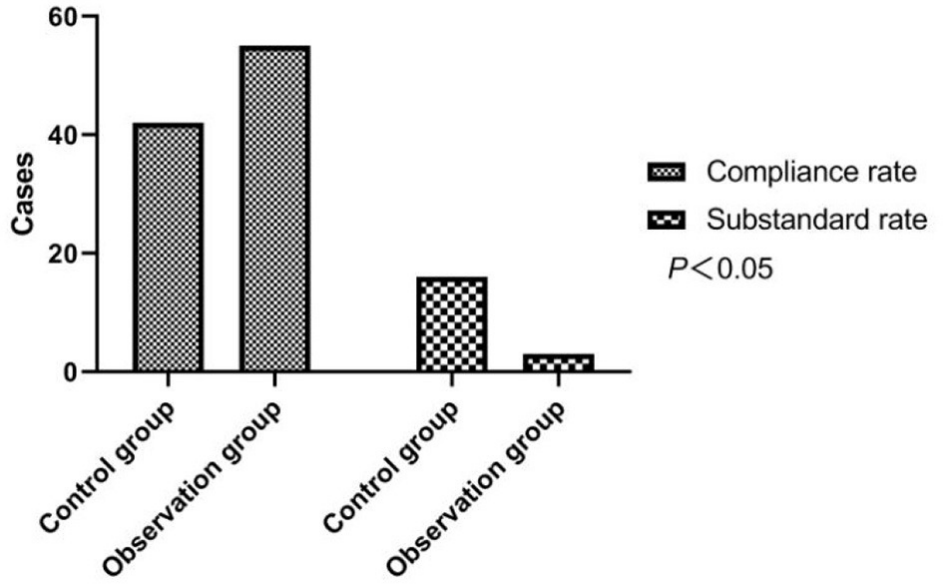
Comparison of health knowledge mastery between two groups of patients.

The control group had 42 adequate cases and 16 inadequate cases, and the observation group had 55 adequate cases and 3 inadequate cases. In comparison with the adequate rate (72.41%) of the group with routine care, strong evidence of a higher adequate rate (94.83%) was found in the group with the continuity of care intervention, with χ^2^ = 10.637 and *p* < 0.05. As shown in Figure 1.

### Comparison of the Self-Care Ability of the Two Groups of Patients

The patients after the intervention showed a higher outcome in all dimensions and the total scores of ESCA (*p* < 0.05). As shown in Table 2.

**Table 2:** Comparison of Exercise of Self-Care Agency (ESCA) scores between the two groups of patients (x̄ ± s, points).

**Groups**	**Self-concept**	**Self-responsibility**	**Self-care skills**	**Health knowledge level**	**Total scores**
Control group (*n* = 58)	19.37 ± 3.24	18.52 ± 3.08	23.78 ± 3.26	41.39 ± 4.52	103.28 ± 10.15
Observation group (*n* = 58)	23.54 ± 4.10	21.69 ± 4.13	27.31 ± 4.58	50.03 ± 4.37	126.54 ± 11.48
*t*	6.077	4.686	4.782	10.470	11.560
*p*	<0.001	<0.001	<0.001	<0.001	<0.001

### Comparison of Medication Compliance Between the Two Groups of Patients

Table 3 revealed that there had been a marked increase in the compliance rate after the intervention in the observation group, with a rate of 96.55% of the observation group vs. a rate of 82.76% of the control group (*p* < 0.05).

**Table 3:** Comparison of medication compliance between the two groups of patients [*n* (%)].

**Groups**	**Complete compliance**	**Partial compliance**	**Non-compliance**	**Overall compliance rate**
Control group (*n* = 58)	25 (43.10)	23 (39.66)	10 (17.24)	48 (82.76)
Observation group (*n* = 58)	39 (67.24)	17 (31.03)	2 (3.45)	56 (96.55)
χ^2^				5.949
*p*				0.015

### Comparison of the Quality of Life of the Two Groups of Patients

The two groups did not differ in terms of all aspects of the quality of life of the patients before the intervention (*p* > 0.05). However, a greater augment in the quality of the life of the patients has been witnessed after the intervention in the observation group, as compared to the control group (*p* < 0.05). As shown in Table 4.

**Table 4:** Comparison of MOS 36-item short-form health survey (SF-36) scores in each dimension between the two groups of patients (x̄ ± s, points).

**Dimension**	**Before intervention**			**After intervention**		
	**Control group (*n* = 58)**	**Observation group (*n* = 58)**	***t***	***p***	**Control group (*n* = 58)**	**Observation group (*n* = 58)**	***t***	***p***
PF	72.54 ± 10.12	71.75 ± 10.46	0.413	0.680	79.17 ± 10.62	85.64 ± 10.77	3.258	0.002
RP	64.18 ± 8.93	65.23 ± 9.04	0.629	0.530	69.36 ± 9.41	75.59 ± 10.58	3.351	0.001
BP	64.15 ± 10.01	64.05 ± 9.73	0.055	0.957	68.59 ± 10.27	76.35 ± 10.92	3.942	<0.001
GH	51.69 ± 10.17	52.31 ± 9.45	0.340	0.734	58.25 ± 10.64	64.83 ± 11.16	3.250	0.002
VT	48.92 ± 7.35	47.63 ± 7.15	0.958	0.340	52.87 ± 8.05	61.27 ± 9.46	5.150	<0.001
SF	49.12 ± 8.15	50.07 ± 8.34	0.620	0.536	57.36 ± 9.11	66.89 ± 9.83	5.415	<0.001
RE	54.71 ± 9.04	53.91 ± 9.13	0.474	0.636	61.85 ± 9.65	72.54 ± 11.05	5.549	<0.001
MH	43.19 ± 8.68	44.28 ± 8.57	0.681	0.498	53.43 ± 9.25	65.49 ± 10.97	6.401	<0.001

### Comparison of the Mental State of the Two Groups of Patients

Before the intervention, no significant difference in the SAS and SDS scores of the two groups was evident (*p* > 0.05). Nevertheless, as shown in Table 5, the observation group obtained a greater drop of SAS and SDS scores in contrast to the control group (*p* < 0.05).

**Table 5:** Comparison of Self-Rating Anxiety Scale (SAS) and Self-Rating Depression Scale (SDS) scores between the two groups of patients (x̄±s, points).

**Groups**	**SAS**			**SDS**		
	**Before intervention**	**After intervention**	***t***	***p***	**Before intervention**	**After intervention**	***t***	***p***
Control group (*n* = 58)	53.24 ± 10.11	47.62 ± 9.94	3.019	0.003	55.38 ± 12.67	48.72 ± 12.09	2.896	0.005
Observation group (*n* = 58)	52.71 ± 9.83	40.16 ± 9.01	7.168	<0.001	54.93 ± 12.36	43.05 ± 11.21	5.422	<0.001
*t*	0.286	4.235			0.194	2.619		
*p*	0.775	<0.001			0.847	0.010		

### Comparison of Nursing Satisfaction Between the Two Groups of Patients

**Figure 2:**
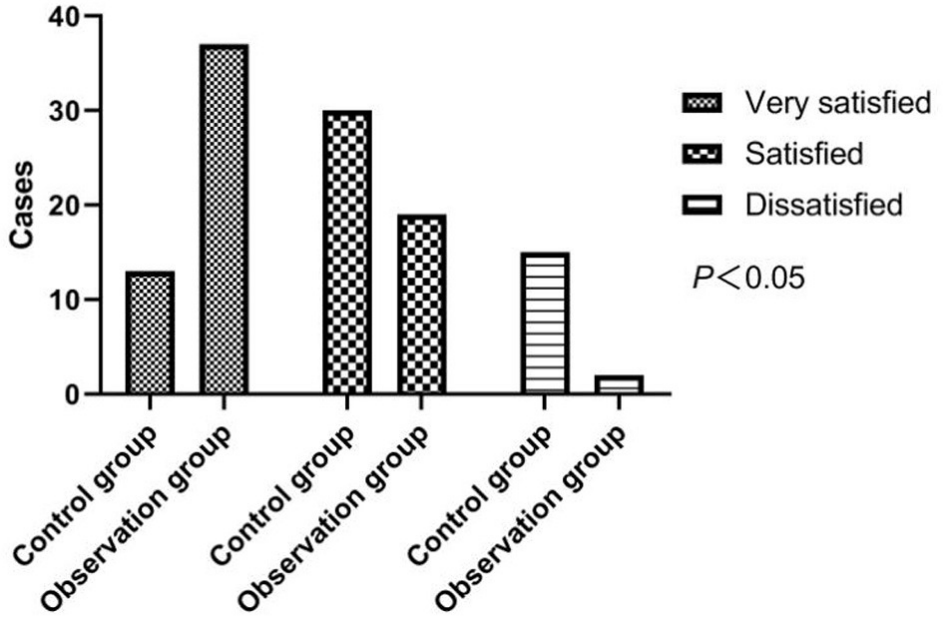
Comparison of nursing satisfaction between two groups of patients.

In the control group, 13 cases were very satisfied, 30 cases were satisfied, and 15 cases were dissatisfied; in the observation group, 37 cases were very satisfied, 19 cases were satisfied, and 2 cases were dissatisfied. After the intervention, a strikingly higher nursing satisfaction rate of 96.55% of the observation group was collected, comparing with the rate of 74.14% in the control group (*p* < 0.05). As shown in Figure 2.

## Discussion

In recent years, changes in the dietary structure of the people, the pace of life, and behavioral habits have brought a rising incidence of PU (17). The causes of PU are mainly gastric acid hypersecretion, *H pylori* infection, and impaired gastric mucosal protection, basically manifested as epigastric pain, acid reflux, heartburn, etc., which takes a toll on the quality of life of the patients (18, 19). The deficiency of disease-related knowledge and self-management ability of some patients with PU often leads to relapses after discharge (20). Therefore, high-quality nursing services should be provided in the middle of treatment. It is, consequently, of great significance for the rehabilitation of patients to organize effective information-based health education and continuity of care after discharge.

In this study, the total effective rate of treatment, ESCA scores in all dimensions, medication compliance rate, SF-36 scores in all dimensions, health knowledge adequate rate, and total nursing satisfaction of patients in the observation group all exceeded those of the control group. It proved that the combined application of information-based health education and continuity of care was effective at ameliorating the therapeutic effect by enhancing the self-care ability of the patients; lifting medication compliance, improving the quality of life of the patients, promoting health-related knowledge and awareness of the disease, and upgrading the quality of care. Compared with the control group, the patients in the observation group had lower SAS and SDS scores, indicating that this method is conducive to the psychological states of the patients such as anxiety and depression elimination. The reminding from the nursing staff and telephone follow-up once a week and home follow-up once a month emphasized to patients the importance of taking medication on time; information-based health education allowed patients to learn various knowledge about PU, helped them realize the importance of timely and correct medication for recovery. As a result, the patients were more positive toward the treatment, and curative effectiveness was significantly elevated along with the medication compliance (21). With the widespread use of smartphones, most people are reluctant to spend time reading paper health readings. The reasonable use of WeChat public accounts and short videos with various forms not only allowed patients to learn health knowledge but also save time both for patients to read and in promoting disease-related health knowledge to patients. During the follow-up, the nursing staff could teach the patients self-care skills on the spot, and correct the wrong nursing methods of the patients, to ensure a better self-care ability of the patients. The patients were more satisfied with the nursing because the gentle attitude, smiles, and considerate services of the staff won the recognition of the patients. The active communication with patients helped the nursing staff fully understand the mental state of the patients, eliminate the inner fear of the disease of the patients, and encourage the patients to maintain an optimistic attitude, which yielded a positive outcome in building up the confidence in the treatment of the patients and improving the psychological state of the patients. It also established a trusting nurse-patient relationship, which was conducive to the compliance with treatment of the patients. After the patients received a series of high-quality care, the ulcer surface quickly healed, and the mental state of the patients was improved, so the quality of life has also been greatly elevated (22). The limitation of this study is that there is no long-term follow-up, which fails in obtaining the long-term clinical efficacy. Moreover, this study did not collect the follow-up data for information-based health education combined with the continuity of care, which will be further extended in future studies to obtain more clinical data.

In conclusion, information-based health education combined with the continuity of care can upgrade clinical efficacy, medication compliance, and nursing satisfaction, enhance disease cognition and self-care ability and elevate the quality of life and mental state. It is of application value in clinical practice.

## Data Availability Statement

The original contributions presented in the study are included in the article/supplementary materials, further inquiries can be directed to the corresponding author/s.

## Ethics Statement

The studies involving human participants were reviewed and approved by Jiaozhou Central Hospital of Qingdao. The patients/participants provided their written informed consent to participate in this study.

## Author Contributions

All authors listed have made a substantial, direct and intellectual contribution to the work, and approved it for publication.

## Conflict of Interest

The authors declare that the research was conducted in the absence of any commercial or financial relationships that could be construed as a potential conflict of interest.

## Publisher's Note

All claims expressed in this article are solely those of the authors and do not necessarily represent those of their affiliated organizations, or those of the publisher, the editors and the reviewers. Any product that may be evaluated in this article, or claim that may be made by its manufacturer, is not guaranteed or endorsed by the publisher.
